# Martha Jane Zachert, PhD, AHIP, FMLA

**DOI:** 10.5195/jmla.2018.502

**Published:** 2018-07-01

**Authors:** Jane Bridges

**Affiliations:** Tybee Island, GA

Martha Jane Koontz Zachert, AHIP, FMLA, retired professor emerita, died January 10, 2018, in Tallahassee, Florida, where she had been retired for a number of years. She was born in York, Pennsylvania, and grew up the daughter of a minister in Baltimore, Maryland. She received a bachelor’s degree from Lebanon Valley College, her master of library science degree (MLS) from Emory University, and her doctorate in library science from Columbia University. In 1946, Martha Jane married Edward Goneke Zachert (nicknamed Zach) with whom she moved to Atlanta, Georgia. They had one daughter, Beth.

**Figure:**
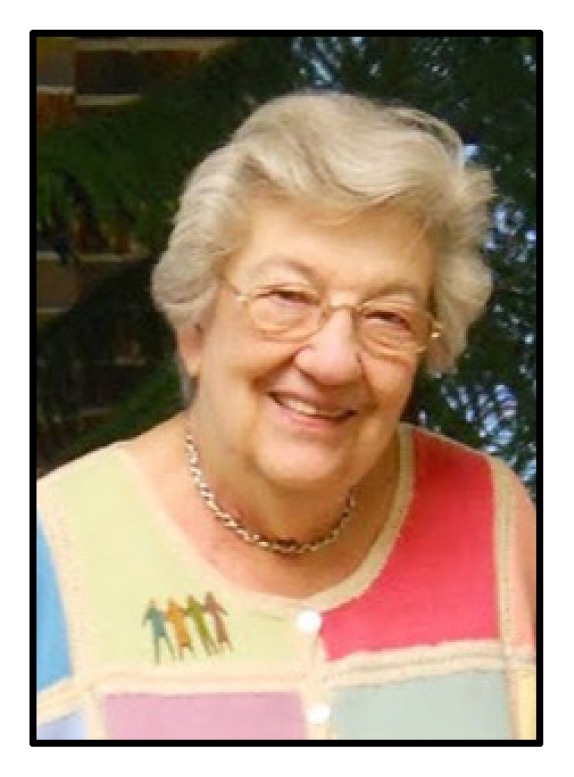


**Figure 1:**
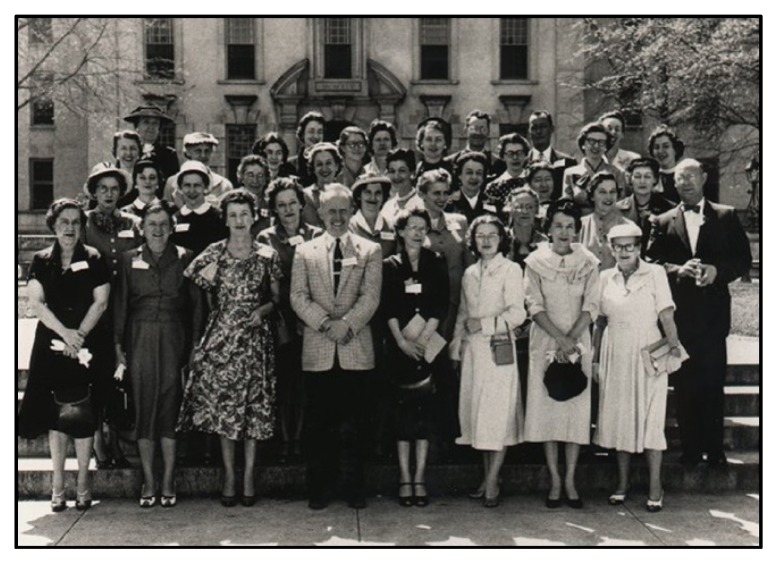
1956 photo of members of the Southern Regional Group, predecessor to the Southern Chapter of the Medical Library Association

Her library career began in 1941 at Enoch Pratt Free Library in Baltimore and continued in schools in DeKalb County, Georgia. She transitioned into medical librarianship at the Mercer University College of Pharmacy in Atlanta and eventually held faculty positions at the library schools of Florida State University and the University of South Carolina. Her secondary teaching field was related to the book arts and history of books. She was a visiting fellow of the British Library and worked with the Army Corps of Engineers library system. She was a founding member of the Southern Regional Medical Library Group, which later became the Southern Chapter of the Medical Library Association (MLA) (Figure 1).

She was an MLA Fellow and named among the “100 Most Notables” during MLA’s Centennial Celebration in 1998. She was a member of the American Library Association, member of Beta Phi Mu and president of Beta Phi Mu in 1974–1975, member of the Special Libraries Association (SLA) and past president of the SLA Florida chapter, and member of the Oral History Association. Among other honors, Martha Jane received the SLA President’s Citation in 1977 and was inducted into the SLA Hall of Fame in 1985, and she received citations from the American Association of Colleges of Pharmacy and the Southeastern Library Association (SELA), including the Rothrock Award, SELA’s highest. She published numerous articles and books in her field, and edited the *Journal of Library History* and several books. She taught short courses in England several times and served six months as consultant to a research project of the British Library.

She held leadership positions related to professional continuing education and was an invited speaker at numerous library meetings, including the 1978 Janet Doe Lecture at MLA, “Books and Other Endangered Species: An Inquiry into the Values of Medical Librarianship.” She certainly had the background for it, having been a member of the American Printing History Association and a founding member of the Miniature Book Society in addition to her Georgia Division of Archives certificate in archival management. In 2015, a former student, Elaine Crepeau—with her husband, Nobuo Kodama—expressed her admiration and gratitude to Martha Jane by establishing the Dr. Martha Jane Zachert Endowed Scholarship at Florida State University.

Several years after I graduated, she made a presentation at a library conference that I attended. I had taken my graduate diploma with me and asked her to sign it. Her signature is as neat as if it were signed on a line, which it was not. That signature means much more to me than the governor’s or the university president’s.

She was honored for her exemplary professional work, but those who knew her personally described the personal gifts she brought to the profession:

Fred W. Roper, AHIP, FMLA:

She was a true Renaissance woman. She worked hard and produced a lot because of her diverse interests.

Ted Srygley:

I was always in awe of her professionally. She made a difference with all the students she taught and the example she set.

Priscilla L. Stephenson, AHIP:

I never heard anything but praises for this remarkable woman. She’s an icon in our world.

Diane Rourke, AHIP:

Her teaching was engaging and kind of magical. You never knew you were learning while you were listening and yet, you were. Not many library school professors were that compelling and dynamic. She also kept sharing her knowledge, particularly on how to teach, as our librarian role expanded to include more teaching.

M.J. Tooey, AHIP, FMLA:

I am so saddened by this news. Just recently, I found some notes from her regarding a project she was helping me with. She was a kind soul and a great help to me when I was a young librarian.

Sarah H. Gable, AHIP:

What I remember is what a great teacher she was. She used role playing techniques to get the point across in an interesting way. She was interested in and supportive of all types of libraries, and encouraging to professionals.

In retirement, Martha Jane returned to Tallahassee, where she became active at St. John’s Episcopal Church and in the Tallahassee Literary Club, while enjoying time with her daughter’s and granddaughter’s families and her sister Miriam Drucker (who predeceased her). She traveled as long as Zach—the love of her life, constant companion, and support—was able. He predeceased her in 2005. Martha Jane was capable of great love for her family, friends, colleagues, and her profession, and was a tremendous personal and professional resource. She will be long remembered.

